# Chorioretinal Atrophy Growth After Voretigene Neparvovec Retinotopically Is Connected to Retinal Functional Rescue

**DOI:** 10.1167/tvst.13.2.13

**Published:** 2024-02-20

**Authors:** Krunoslav Stingl, Melanie Kempf, Ronja Jung, Katarina Stingl

**Affiliations:** 1 University Eye Hospital, Center for Ophthalmology, University of Tuebingen, Tuebingen, Germany; 2 Center for Rare Eye Diseases, University of Tuebingen, Tuebingen, Germany

**Keywords:** rpe65, voretigene neparvovec, retinitis pigmentosa, retinal metabolism, gene therapy

## Abstract

**Purpose:**

Chorioretinal atrophy growth after voretigene neparvovec has been reported recently with its positive correlation with successful treatment. This finding raised the question on long-term effects and the etiology of the chorioretinal atrophy.

**Methods:**

Using local retinal functional diagnostics, we tested whether the atrophy growth is connected to the initial local functional improvement after the therapy.

**Results:**

The results describe factors predicting the development of atrophy. First, the atrophy emerges after approximately 3 months in an area with local functional rescue before. The areas of the greatest gain in the number of functionally rescued rods are prone to be the initial spots of atrophy growth in almost one-half of the cases and the retinotopy corresponds with the area of a high number of post-treatment functioning rods. Second, the dark-adapted perimetry shows that the atrophy growth is in the area with functioning rescued rods. However, the rods with the greatest sensitivity gain are not the parts of the growing atrophy in the first 2 years after intervention. This preservation of rods with the greatest sensitivity seems to explain the excellent profile of rods rescue over the long term measured by full-field stimulus threshold and reported earlier.

**Conclusions:**

A disbalance between the increase of functional rods and their threshold shortly after treatment could be an indicator for a metabolic origin of chorioretinal atrophy after voretigene neparvovec.

**Translational Relevance:**

A basic understanding of the photoreceptor rescue aspects after gene therapy can demonstrate a metabolic causal influence of the efficacy on the development of side effects, such as chorioretinal atrophy.

## Introduction

Voretigene neparvovec as the first approved gene supplementation therapy for *RPE65* retinal dystrophies has been approved for clinical use in 2017 and 2018. The phenotype of the RPE65 retinal dystrophy is mostly an early onset retinal dystrophy with a rapid progression of degeneration leading to blindness in young or middle adult age.^1^^,^^2^ Voretigene neparvovec is the only causal treatment for inherited retinal dystrophies with well documented functional rods rescue.^3^^,^^4^ It has been shown repeatedly that age is one of the most important factors influencing the treatment outcome, with younger patients having more functional benefit than patients with more progressed degeneration.^4^^,^^5^ A phase III clinical trial showed that voretigene neparvovec has an acceptable safety profile^3^; however, chorioretinal atrophy growth, mostly around the vessel arcades and in the middle periphery, has been reported after approval by several recent studies.^5^^–^^7^ There are probably several reasons why no signs of this phenomenon had been reported before approval. First, fast developing retinal imaging, including fluorescein autofluorescence, introduced the possibility of better and precise atrophy mapping. Second, the position of the atrophy, being usually in the middle periphery, did not seem to directly affect psychophysiological primary endpoints of the clinical trials for voretigene neparvovec. Nevertheless, recent reports, for example, from our group and the Children’s Hospital in Los Angeles, revealed that these atrophies are not a rare event. A rough estimate indicates that almost 30% of treated patients will develop this fast-progressing atrophic changes.^5^^,^^7^^,^^8^ The available data show that the origin is not driven by a specific on-site surgical procedure.^5^ Data from these two major sites for gene therapy revealed that the profile of subjects developing growing chorioretinal atrophy is correlated with high rod rescue benefit after therapy,^5^ affecting predominantly younger subjects. Interestingly, an exception from this correlation was seen if patients were below a certain age: in preschool children, the fast-growing chorioretinal atrophy was usually not seen, despite or independent of good functional benefit after treatment.^5^

Based on these results, we could hypothesize that the high functional rod rescue leads to increased metabolic demand or toxic effects, resulting thus in an accelerated outer retina atrophy development. However, very young children do not fit into this scenario; the hypothesis that patients who react well to treatment do develop growing chorioretinal atrophy is, thus, not correct.

Instead, an additional explanation might be valid: young patients—such as teenagers and young adults—benefit from a good rod rescue by this gene therapy, but their retina has reached some stage of degeneration before treatment. This factor leads to a metabolic or oxidative imbalance in the tissue owing to suddenly increased rod function and metabolic turnover. Such an imbalance could be the actual reason for the observed development of atrophy. However, the retinas of very young children, who also have good functional rescue after treatment, are still better preserved and thus can adapt to the increased metabolism. For that reason, chorioretinal atrophies after voretigene neparvovec in preschool children might be rare. This observation can be important or lead to a hypothesis that illustrates that the therapy effect and safety can be related. Still, these hypotheses, although based on already published data, need further analysis.

The goal of this work was to explore possible mechanisms of the atrophy growth based on local retinal functional (especially for rods) and imaging data of patients treated with voretigene neparvovec at the Center for Ophthalmology at the University of Tuebingen.

The functional benefit of voretigene neparvovec on rod function is usually well measurable at month 1 after treatment. Interestingly, the functional rescue at that time point is usually at its maximum, decreases slightly to month 3, but then stays stable over the following months and years.^4^^,^^5^ The chorioretinal atrophies usually develop only after the first functional benefit can be observed, around the third month after the treatment.^6^^,^^7^

Our hypothesis was that the development and growth of the chorioretinal atrophy after voretigene neparvovec is related to the local metabolic state preceding the atrophy growth and that the local post-treatment functional and metabolic state of the retina can be predictive of the atrophy growth. Information on the metabolic turnover of the retinal locations were obtained from local rod functional examinations: dark-adapted chromatic perimetry (DACP), providing the local dark-adapted thresholds, and scotopic chromatic pupil campimetry (sCPC), a dark-adapted readout using the pupil response on local scotopic stimuli related to the number of rescued rods in the corresponding area.^4^^,^^9^^,^^10^

To that end, we addressed the following questions. First, what is the dynamics of the chorioretinal atrophy growth? Second, is the retinal area where the atrophy growth starts at around month 3 a part of the initial treatment response or not? And third, does the area of the maximal local rod rescue become a center for chorioretinal atrophy or is it spared? In other words, we tried to analyze whether the areas of atrophy growth show specific functional rescue pattern before becoming atrophic.

## Methods

Eleven eyes from six subjects with chorioretinal atrophy growth after voretigene neparvovec were retrospectively analyzed with a focus on local functional rescue. Some of the functional retinal data of the first five subjects have been published previously.^4^ Two functional biomarkers of local rod function were analyzed: DACP and sCPC, characterizing different aspects of the rod function. A 30° protocol of DACP was tested after 20 minutes of dark adaptation and describes the local rod threshold sensitivity. The protocol of the sCPC was performed in a dark-adapted state as well, using 41 local scotopic stimuli inside of the 30° visual field presented on a wide-screen monitor. By means of an infrared camera, the pupil response to the local light stimulation was analyzed. Because the pupil response is driven by the numbers of stimulated photoreceptors connected to the reflex pathway, the pupil response amplitude of the sCPC can be considered as an indirect correlate of the local number of rods functioning in the scotopic range matching the retinotopy of the stimulus. Both rod protocols have been validated for the evaluation of local retinal rod function and have been described in detail previously.^11^

Retinotopic maps of these readouts were created at month 1 after treatment, because this time point represents usually the highest functional rod rescue.^4^^,^^9^

For retinal imaging, fundus autofluorescence (FAF) data (30° image, Heidelberg Engineering GmbH, Heidelberg, Germany) at month 3 were used, as the atrophy growth usually starts around the third month.^5^^–^^7^ The delineation of outer retinal atrophy lesions is well possible with FAF imaging, even though the autofluorescence is reduced in the *RPE65* phenotype.^9^^,^^12^ An experienced ophthalmologist with expertise in inherited retinal degenerations manually marked all the FAF images for definition of chorioatrophic areas. The percentage of the atrophic areas in the entire 30° FAF image was calculated for each subject at each time point after treatment.

Functional maps acquired from DACP and sCPC (at month 1) were then superimposed on the FAF images (at month 3). Thereafter, the maximal value from these functional measurement at month 1 in the atrophic and in the nonatrophic areas were extracted for both DACP and sCPC. In this way, we tried to explore whether the atrophic areas at month 3 matched those areas that responded to therapy at month 1 and whether the affected retinotopy also corresponds with the area of the maximal rescue. Owing to the relatively small cohort of subjects with high heterogeneity and statistical dependency between both eyes, we present the data in a descriptive way.

## Results

**Figure 1.**
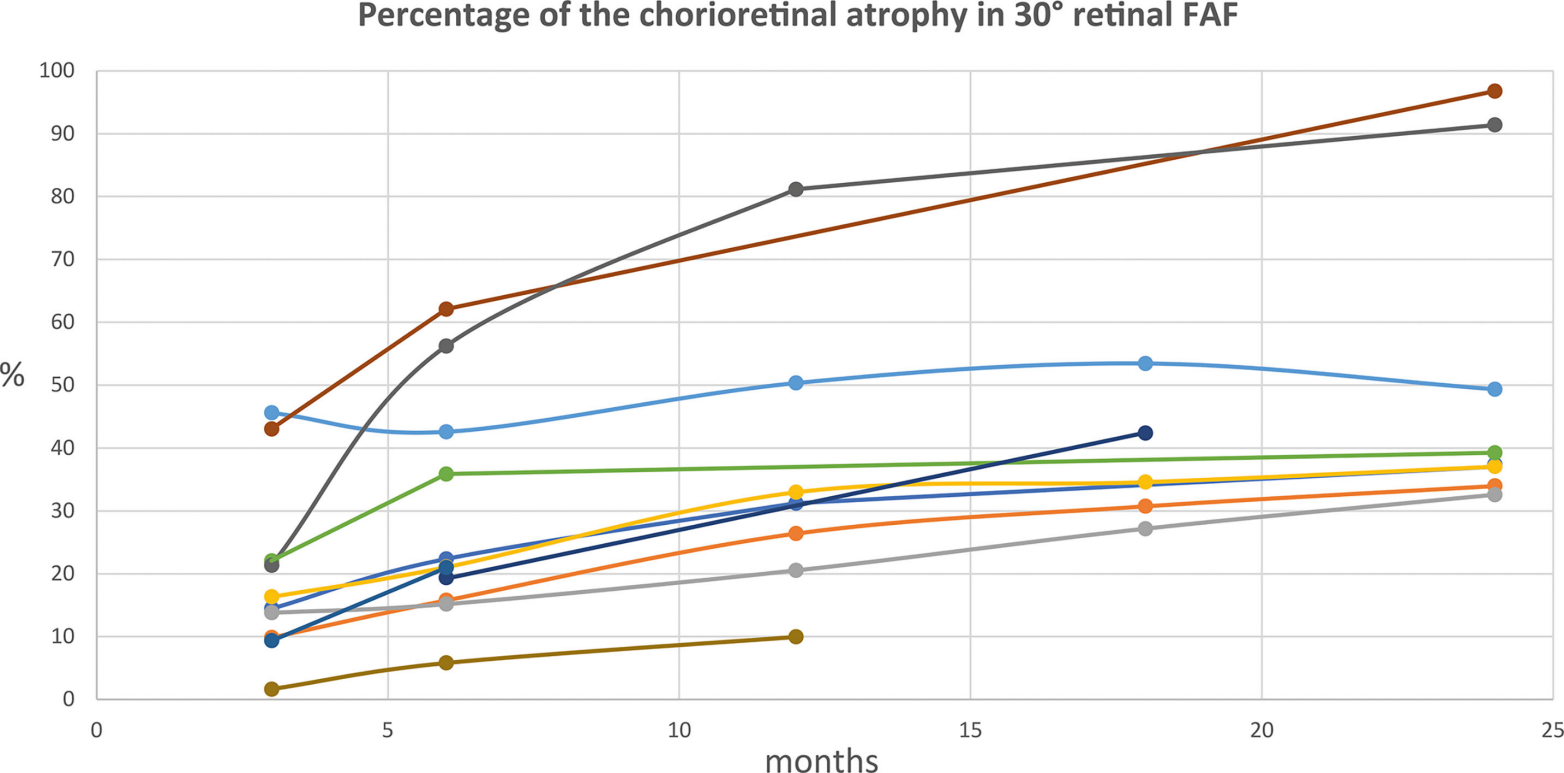
Size growth of the chorioretinal atrophies inside of the 30° FAF image over 24 months after the treatment in all eyes.

In all 11 eyes, the chorioretinal atrophy grew over 24 months after surgery, as measured using the area inside of the 30° autofluorescence image (Fig. 1). The baseline characteristic of the patients at the time of the treatment, such as age, best-corrected visual acuity and full-field stimulus threshold (FST) values for blue and red stimuli are summarizes in Table 1.

**Table 1.** Baseline Characteristics of Patients at the Time of Treatment

	Age at Treatment, Years	BCVA Baseline	BCVA Month 1	FST Blue Baseline, dB	FST Blue Month 1, dB
01 LE	21	20/125	20/125	−9.71	−18.84
01 RE	21	20/160	20/160	−0.83	−26.20
03 LE	14	20/320	20/200	−0.72	−44.00
03 RE	15	20/50	20/50	−19.51	−28.10
08 LE	23	20/100	20/100	−7.60	−34.73
10 LE	17	20/160	20/80	−0.80	−26.38
10 RE	17	20/100	20/100	−3.13	−13.63
12 LE	33	HM	HM	1.02	2.46
12 RE	33	HM	HM	2.64	0.57
14 LE	8	20/400	20/200	1.78	−19.80
14 RE	9	20/160	20/125	−3.13	17.30

BCVA, best-corrected visual acuity.

The FST values are reported in dB with 0dB set to 0.01 cd/m^2^.

**Figure 2.**
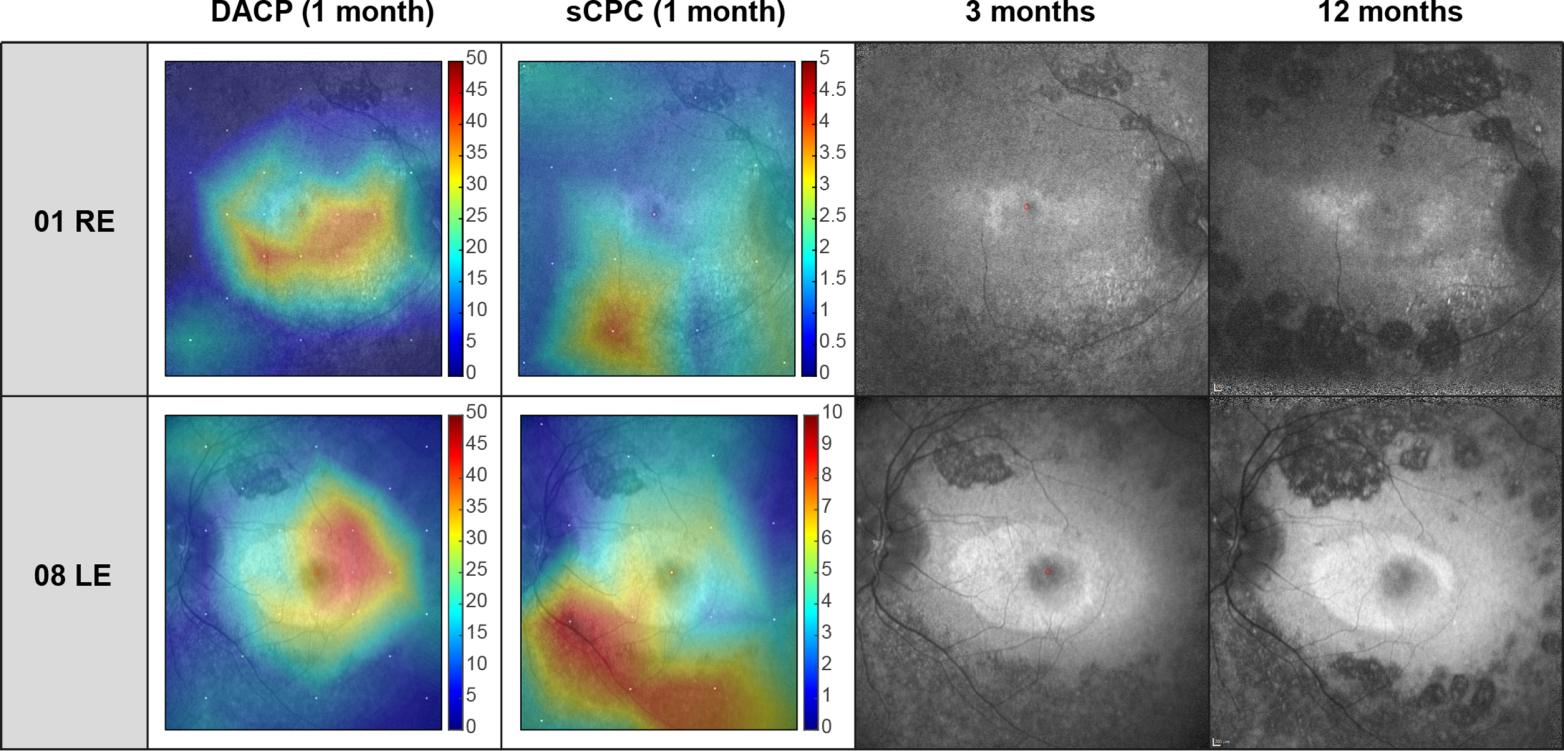
Superimposed functional maps of the sCPC and DACP from month 1 onto the retinal imaging from month 3. The FAF images on the right illustrate the fast development of the chorioretinal atrophy between month 3 and month 12 for the corresponding eyes. Both patients demonstrate cases, for whom the area of the initial rod threshold improvement (DACP sensitivity maps month 1) does not involve the areas of later chorioretinal atrophy growth, but the areas of initial CPC rescue (increase in number of functional rods in month 1) correspond with the chorioretinal atrophy growth. The response values of the relative pupil constriction in healthy subjects for central 30° for sCPC is between 15 (central) and 10 (peripheral); and for DAC around −60 dB, as published in Stingl et al.^11^

Superimposed functional maps of the sCPC and DAC from month 1 onto the retinal imaging from month 3 are shown in Figure 2, as well as the FAF images of the corresponding month 3 and month 12 to illustrate the regions of fast chorioretinal atrophy development. For comparison, normal values for DAC values and sCPC can be found in Stingl et al.^11^

### Analysis of the Predictive Value of the sCPC

In the cohort of 11 eyes, a clear rod rescue in sCPC at month 1 was observed in 9 eyes (all except both eyes of subject 10) (Table 1). A clear rescue was defined as a measurable pupil constriction of at least 1% in at least one stimulus location.

**Figure 3.**
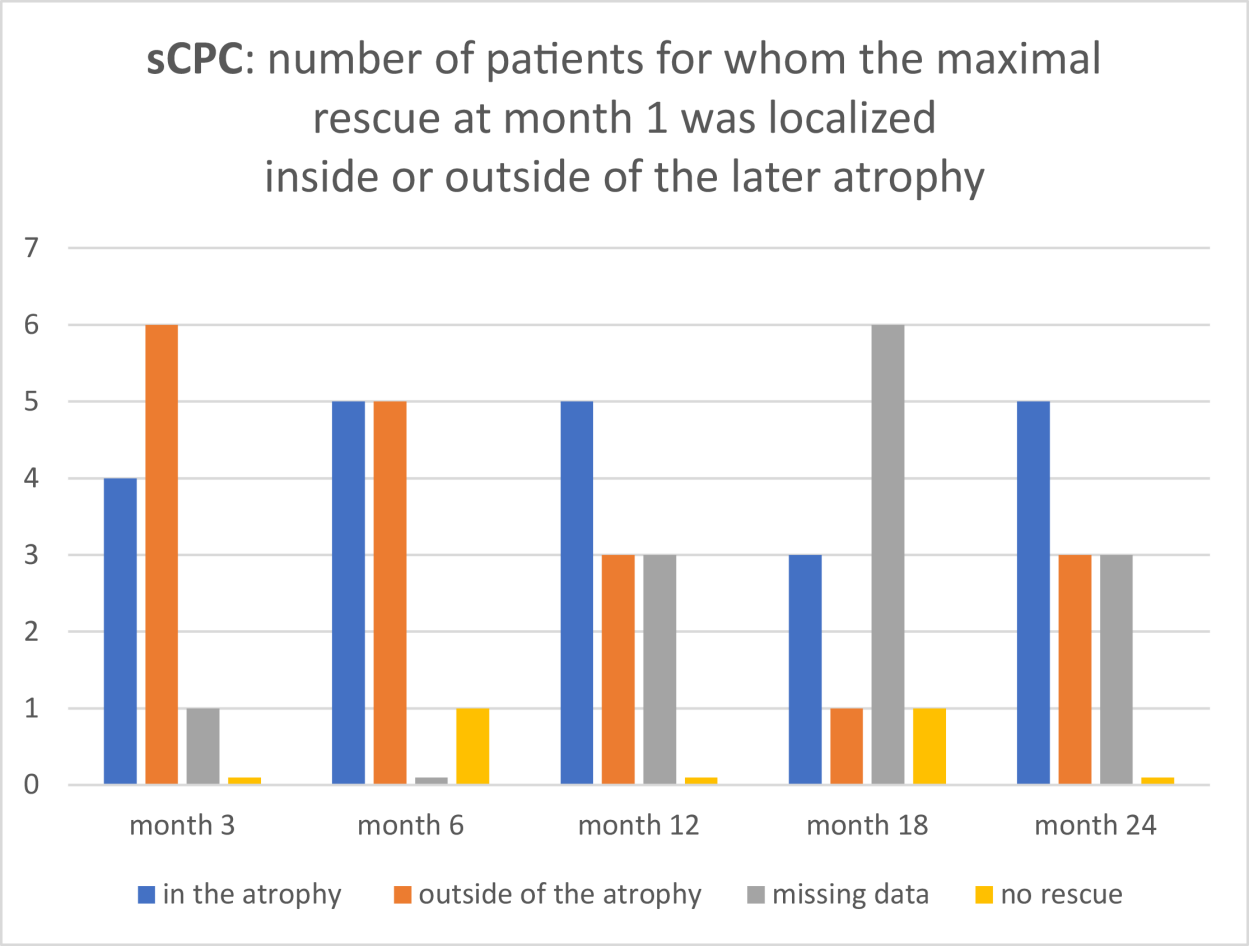
Histogram of number of cases whose maximal month 1 response in sCPC was localized inside or outside the later developing atrophy. The figure is a graphical representation of the result data from Table 1.

In seven eyes, the 3-month-atrophy developed in a retinotopy that had a functional rescue measured by CPC at month 1 (01–right eye [RE], 03–left eye [LE], 03–RE, 08–LE, 12–LE, 12–RE, and 14–RE) (Table 1). In four eyes, the 3-month atrophy area covered the highest rod rescue measured at month 1 (01–RE, 03–RE, 08–LE, and 12–LE) (Fig. 2, Table 2, Fig. 3^4^, blue bars). At month 24, the maximal 1-month rod rescue was inside of the atrophic area in five eyes (01–RE, 03–RE, 08–LE, 12–LE, and 12–RE) (Table 2, Fig. 3, blue bars).

**Table 2.** Maximal Local Values of the sCPC

sCPC	Maximal Rescue at Month 1 in the 30° Area	Maximal Rescue at Month 1 in the Month 3 Atrophy Area	Maximal Rescue at Month 1 in the Month 3 Nonatrophy Area	Maximal Rescue at Month 1 in the Month 6 Atrophy Area	Maximal Rescue at Month 1 in the Month 6 Nonatrophy Area	Maximal Rescue at Month 1 in the Month 12 Atrophy Area	Maximal Rescue at Month 1 in the Month 12 Nonatrophy Area	Maximal Rescue at Month 1 in the Month 18 Atrophy Area	Maximal Rescue at Month 1 in the Month 18 Nonatrophy Area	Maximal Rescue at Month 1 in the Month 24 Atrophy Area	Maximal 1 Rescue at Month 1 in the Month 24 Nonatrophy Area
01 LE	1.3^a^	0.6^b^	1.3^c^	0.7^b^	1.3^c^	0.7^b^	1.3^c^	n.a.^b^	n.a.^c^	0.8^b^	1.3^c^
01 RE	3.0^a^	3.0^b^	2.9^c^	3.0^b^	2.8^c^	3.0^b^	2.5^c^	3.0^b^	2.5^c^	3.0^b^	2.3^c^
03 LE	7.4^a^	4.8^b^	7.4^c^	4.7^b^	7.4^c^	5.4^b^	7.4^c^	5.8^b^	7.4^c^	6.2^b^	7.4^c^
03 RE	3.5^a^	3.5^b^	2.9^c^	3.5^b^	2.9^c^	3.5^b^	2.9^c^	3.5^b^	2.9^c^	3.5^b^	2.9^c^
08 LE	6.8^a^	6.8^b^	6.6^c^	6.8^b^	6.5^c^	6.8^b^	6.5^c^	6.8^b^	6.5^c^	6.8^b^	6.6^c^
10 LE	0.9^a^	0.5^b^	0.9^c^	0.5^b^	0.9^c^	n.a.^b^	n.a.^c^	n.a.^b^	n.a.^c^	0.5^b^	0.9^c^
10 RE	0.0^a^	n.a.^b^	n.a.^c^	0.0^b^	0.0^c^	n.a.^b^	n.a.^c^	0.0^b^	0.0^c^	n.a.^b^	n.a.^c^
12 LE	3.8^a^	3.8^b^	3.4^c^	3.8^b^	3.6^c^	3.8^b^	3.6^c^	n.a.^b^	n.a.^c^	3.8^b^	2.9^c^
12 RE	1.7^a^	1.3^b^	1.7^c^	1.7^b^	1.7^c^	1.7^b^	1.6^c^	n.a.^b^	n.a.^c^	1.7^b^	1.2^c^
14 LE	1.3^a^	0.0^b^	1.3^c^	0.2^b^	1.3^c^	0.2^b^	1.3^c^	n.a.^b^	n.a.^c^	n.a.^b^	n.a.^c^
14 RE	1.6^a^	1.2^b^	1.6^c^	1.2^b^	1.6^c^	n.a.^b^	n.a.^c^	n.a.^b^	n.a.^c^	n.a.^b^	n.a.^c^

n.a., not available.

a Measured in the relative pupil response on the local stimulus as measured 1 month after the treatment.

b Maximal local value inside the atrophy area at months 3, 6, 12, 18, and 24.

c Maximal local values outside of the atrophy area at months 3, 6, 12, 18, and 24.

**Figure 4.**
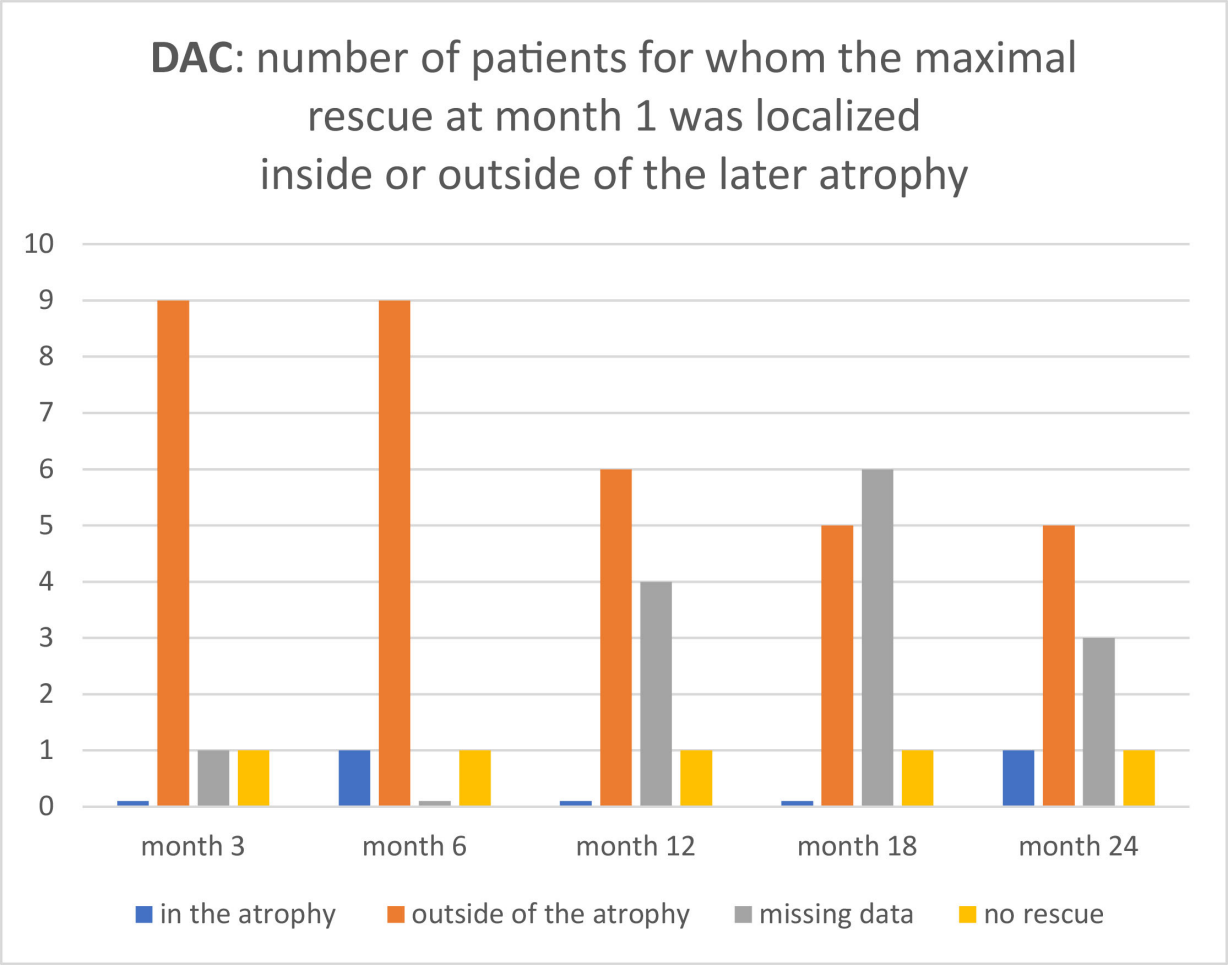
Histogram of number of cases whose maximal month 1 response in DAC was localized inside or outside the later developing atrophy. The figure is a graphical representation of the result data from Table 2.

### Analysis of the Predictive Value of DACP

In the cohort of 11 eyes, a clear rod rescue in DACP at month 1 was observed in 10 eyes (all except 12–LE). A clear improvement was defined as at least one stimulus location with at least 6 dB improvement of the dark-adapted threshold.^11^ In 8 eyes the 3-month-atrophy developed in a retinotopy that had functional rescue measured by DAC at month 1 (all except 10–RE, 12–RE, and 12–LE) (Table 3). None of the eyes had the highest month 1 DACP improvement inside of an atrophic area at month 3 (Fig. 2, Table 3, Fig. 4, blue bars). At month 24, one eye (10–LE) had the maximal month 1 DACP improvement inside of atrophic area (Table 3, Fig. 4, blue bars).

**Table 3.** Maximal Local Values of the DACp

DACP	Maximal Rescue at Month 1 in the 30° Area	Maximal Rescue at Month 1 in the Month 3 Atrophy Area	Maximal Rescue at Month 1 in the Month 3 Nonatrophy Area	Maximal Rescue at Month 1 in the Month 6 Atrophy Area	Maximal 1 Rescue at Month 1 in the Month 6 Nonatrophy Area	Maximal Rescue at Month 1 in the Month 12 Atrophy Area	Maximal Rescue at Month 1 in the Month 12 Nonatrophy Area	Maximal Rescue at Month 1 in the Month 18 Atrophy Area	Maximal Rescue at Month 1 in the Month 18 Nonatrophy Area	Maximal Rescue at Month 1 in the Month 24 Atrophy Area	Maximal Rescue at Month 1 in the Month 24 Nonatrophy Area
01 LE	35.0^a^	22.1^b^	35.0^c^	21.5^b^	35.0^c^	23.1^b^	35.0^c^	n.a.^b^	n.a.^c^	24.9^b^	35.0^c^
01 RE	41.0^a^	26.0^b^	41.0^c^	23.0^b^	41.0^c^	22.7^b^	41.0^c^	25.4^b^	41.0^c^	25.7^b^	41.0^c^
03 LE	37.0^a^	30.7^b^	37.0^c^	31.2^b^	37.0^c^	33.0^b^	37.0^c^	34.9^b^	37.0^c^	35.3^b^	37.0^c^
03 RE	45.0^a^	40.1^b^	45.0^c^	43.3^b^	45.0^c^	41.2^b^	45.0^c^	41.6^b^	45.0^c^	42.4^b^	45.0^c^
08 LE	36.0^a^	20.7^b^	36.0^c^	21.0^b^	36.0^c^	21.0^b^	36.0^c^	21.0^b^	36.0^c^	21.0^b^	36.0^c^
10 LE	15.0^a^	10.8^b^	15.0^c^	14.4^b^	15.0^c^	n.a.^b^	n.a.^c^	n.a.^b^	n.a.^c^	15.0^b^	14.2^c^
10 RE	19.0^a^	n.a.^b^	n.a.^c^	8.1^b^	19.0^c^	n.a.^b^	n.a.^c^	17.0^b^	19.0^c^	n.a.^b^	n.a.^c^
12 LE	0.0^a^	0.0^b^	0.0^c^	0.0^b^	0.0^c^	n.a.^b^	n.a.^c^	n.a.^b^	n.a.^c^	0.0^b^	0.0^c^
12 RE	7.0^a^	5.7^b^	7.0^c^	7.0^b^	6.6^c^	7.0^b^	6.6^c^	n.a.^b^	n.a.^c^	6.9^b^	7.0^c^
14 LE	28.0^a^	25.7^b^	28.0^c^	26.8^b^	28.0^c^	24.4^b^	28.0^c^	n.a.^b^	n.a.^c^	n.a.^b^	n.a.^c^
14 RE	32.0^a^	30.4^b^	32.0^c^	26.7^b^	32.0^c^	n.a.^b^	n.a.^c^	n.a.^b^	n.a.^c^	n.a.^b^	n.a.^c^

n.a., not available.

a Measured as the gain in threshold to local cyan stimuli in dB as measured 1 month after the treatment.

b Maximal local value inside the atrophy area at months 3, 6, 12, 18, and 24.

c Maximal local values outside of the atrophy area at months 3, 6, 12, 18, and 24.

## Discussion

In this study, we evaluated whether local functional rod rescue correlates with the retinotopy of chorioretinal atrophy growth after voretigene neparvovec. Our results demonstrate that in many patients the atrophy develops in high rod recovery areas, as measured via sCPC, but not the areas of the highest rod threshold improvement as measured with DACP.

Interestingly, areas with the highest increase of the dark-adapted sensitivity (DACP rescue peaks) do not necessarily match the areas of the highest increase of functional rod number (sCPC rescue peaks). We could show that the areas of the largest gain in the dark-adapted sensitivity do not match the locations of chorioretinal atrophy growth. In most of the trials, as well as in the approval clinical trial, the dark-adapted threshold has been tested with FST. The FST gain has been shown to be stable over several years after treatment,^5^ and our results reveal that the area with the strongest improvement in dark-adapted sensitivity is not part of the initial atrophic lesion or its growth over 24 months, could explain the stability of the threshold rescue over long time. Our results confirm that areas that generate the highest FST improvement do not become compromised by atrophic changes in the post-treatment time course. Additionally, the chorioretinal atrophy in most of the patients does not include the central macular or foveal region. This also explains why visual acuity or contrast vision usually remains stable after treatment.

In contrast, the sCPC, which uses the pupil response as a readout of local retinal function and can thus be regarded as an indirect biomarker of the number of local functional rods, showed a different pattern. In approximately one-half of our patients, the greatest rod number increase at month 1 corresponded with the retinotopy where the atrophy started at month 3 and spread until the end of the observation time. This indicates that a high number of recovered rod cells in the treated area might precede the atrophy growth and might be a significant predictor for the chorioretinal atrophy development.

Previously, we published that the chorioretinal atrophy growth is found predominantly in therapy responders.^5^ In that cohort, there was a highly significant difference in treatment efficacy between patients with and without chorioretinal atrophy growth. Although the commonly used readout for treatment success is the dark-adapted threshold FST, here in the current article we could show that it is the local increase of functional rods, but not their sensitivity improvement that precedes the atrophy growth. Obviously, patients with high rescue in sCPC are those with the highest DACP (or FST) gain, but the retinal areas depicting the respective treatment effects do not match entirely. That is why we observe a correlation of atrophy growth with FST,^5^ although here we show that the local threshold improvement is not the driving factor of the atrophy growth.

Building on the results of a previously published article,^5^ we propose an explanation for the driving factors of chorioretinal atrophy growth based on these new observations. The atrophy affects mostly children of school age, teenagers, and young adults, who responded well to the treatment, with the retinal location with the highest increase of active rods being a potential predictor for the chorioretinal atrophy. Patients in advanced disease stages often have no measurable rod rescue and do not present with chorioretinal atrophies. An interesting group of patients are young children below school age, who rarely develop this atrophy. Based on all these observations, we speculate that there are three possible cell-dependent scenarios for the therapy effect.

In the first scenario, the degeneration stage of photoreceptors is very progressed and does not allow a strong treatment benefit owing to the low number of target cells. Those cases, especially patients in a late-stage disease, have barely a functional gain of voretigene neparvovec and rarely atrophy growth.^5^^,^^12^

In the second scenario, the treatment effect is relevant, as seen usually in school children, teenagers, or young adults, but presents a big change in rod activity and metabolic stress in the retina, because the retina has already reached a certain level of degeneration. This sudden increase in rod activity inside of a degenerated retina might turn into a disbalance or overload of nutrient and oxygen consumption. Thereafter, the increased metabolic rate can further compromise the degenerative state of the retinal tissue and can trigger accelerated cell death, which results in a fast chorioretinal atrophy growth as we observe it in the clinical picture. Why this situation occurs mostly in the middle periphery cannot be explained completely, but might be connected to the high rod density.

The third scenario affects patients whose retinas are still in a relatively well-preserved state. For those patients, the treatment does present a large benefit owing to the high preservation of target cells. In contrast, the change of the metabolic situation after rod functional rescue does not yet present an extensive burden in the metabolic turnover and oxygen consumption. Such a little metabolic imbalance can still be compensated by the cells the does not lead to cell death. Indeed, a good treatment rescue without chorioatrophic growth is usually observed in young children. However, the phenotypes in small children are not always identical and there are children with a severe retinal degeneration or Leber congenital amaurosis. Also, later in life, the population is rather heterogeneous regarding the level of retinal degeneration, and that is why some teenagers and young adults develop growing chorioretinal atrophies, while others are spared. Thus, we cannot exclude that other mechanisms may play a role in very young children after voretigene neparvovec. However, it has been observed and published that immunological responses, despite a perioperative cortisone schedule, occur especially in the pediatric population, although usually resolved as integrum.^14^^,^^15^

We could observe that there is a specific time dynamic of both the rod rescue and chorioretinal atrophy growth after voretigene neparvovec. At 14 days and 1 month after surgery, there is almost no sign of the chorioretinal atrophy, except at the injection site, which has a different etiology and is not part of the phenomena described in this article. The first clear sign of chorioretinal atrophy is usually obvious at month 3. In our patients, thereafter a very fast progression of atrophy growth occurs between months 3 and 9 or 12. Later, the atrophy usually grows more slowly. The here presented data indicate that chorioretinal atrophy is developing in the part of the retina which had functional rescue before. The fact that atrophy does not follow immediately after the surgery, and it never (based on our cohort data) grows in the areas of the best functional rescue, in our opinion decreases the probability of immunologic or toxic explanation of this phenomena. All in all, our data are from a small population measured over a short period of time; however, the data suggest very specific time and pattern dynamics, not consistent with the theory of inflammation as a main mechanism of atrophy development.

Our results indicate that the atrophy growth could be a metabolic response to the increased number of active rods in responders. The data presented in this article support the explanation that the region with the greatest increase of rescued rods suffers the strongest metabolic imbalance, leading to outer retina atrophy. In contrast with this finding, areas with the greatest sensitivity gain in available cells, as measured with the dark-adapted threshold, seem to be stable over the long term.

Although our analysis using DACP and sCPC could for the first time provide some explanations of these phenomena, there are several aspects that we could not resolve. For example, we do not know if the hypothesized metabolic stress is more pronounced in the retinal pigment epithelium or rods. The target cells for *RPE65* gene supplementation therapy are the retinal pigment epithelium cells, but we measure therapy success based on the effects on rods. To answer these questions, we would need additional evaluations of the retinal metabolism, such as oxygenation or flavoprotein fluorescence, which is related to mitochondrial stress in vivo.

The results presented in this study also have further limitations. First, we evaluated a rather small number of subjects. Although many patients have received treatment with voretigene neparvovec worldwide, protocols at the sites allowing this type of local rod rescue evaluations are very rare. The currently most important readout acknowledged also by regulatory agencies is the visual benefit in daily lives of the patients; thus, especially psychophysical measurements such as FST, mobility tests, and patient outcome reports are being analyzed after gene therapy. Unfortunately, evaluations that objectively capture local retinal function of rescued cells are not commonly reported. However, to understand the retinal processes behind the safety and efficacy treatment effects, a protocol as described here and by Stingl et al.^9^ would be more suitable. We recommend introducing similar protocols to evaluate retinal function after gene therapy also in other sites, to be able to evaluate local retinal effects of gene therapy in a larger scale. That would allow us to evaluate the scientific significance of the presented findings and to further optimize the treatment procedure.

Currently, only a few years of experience are available with patients who have been treated with the approved gene therapy voretigene neparvovec. Although areas with the greatest sensitivity rescue do not seem to be affected by the chorioretinal atrophy growth, which is important for the long-term benefit of the patients, our data currently cannot predict whether the chorioretinal atrophy growth will not involve important retinal areas in the long-term future. This work presents an additional indication that choosing a proper clinical evaluation protocol after gene therapy is crucial for understanding its effect even after approval in a long-term scale.
